# Supplementary material for: A View on the Chemical and Biological Attributes of Five Edible Fruits after Finishing Their Shelf Life: Studies on Caco-2 Cells
Source: Int J Mol Sci. 2024 Apr 29;25(9):4848. doi: 10.3390/ijms25094848 (PMC11084482; doi:10.3390/ijms25094848)

Figure S1 (a-e). Chemiluminescence reaction (CL) along the 60 seconds total time reaction development for the five series of the 50% ethanolic extracts in the study (a.EES, b.EEC, c.EEA, d.EEP, e.EEG), at the beginning (t=0) and at the end of experiment (t=5/7), in comparison the with negative control sample series (the green line); n=3; ± SD (1%).

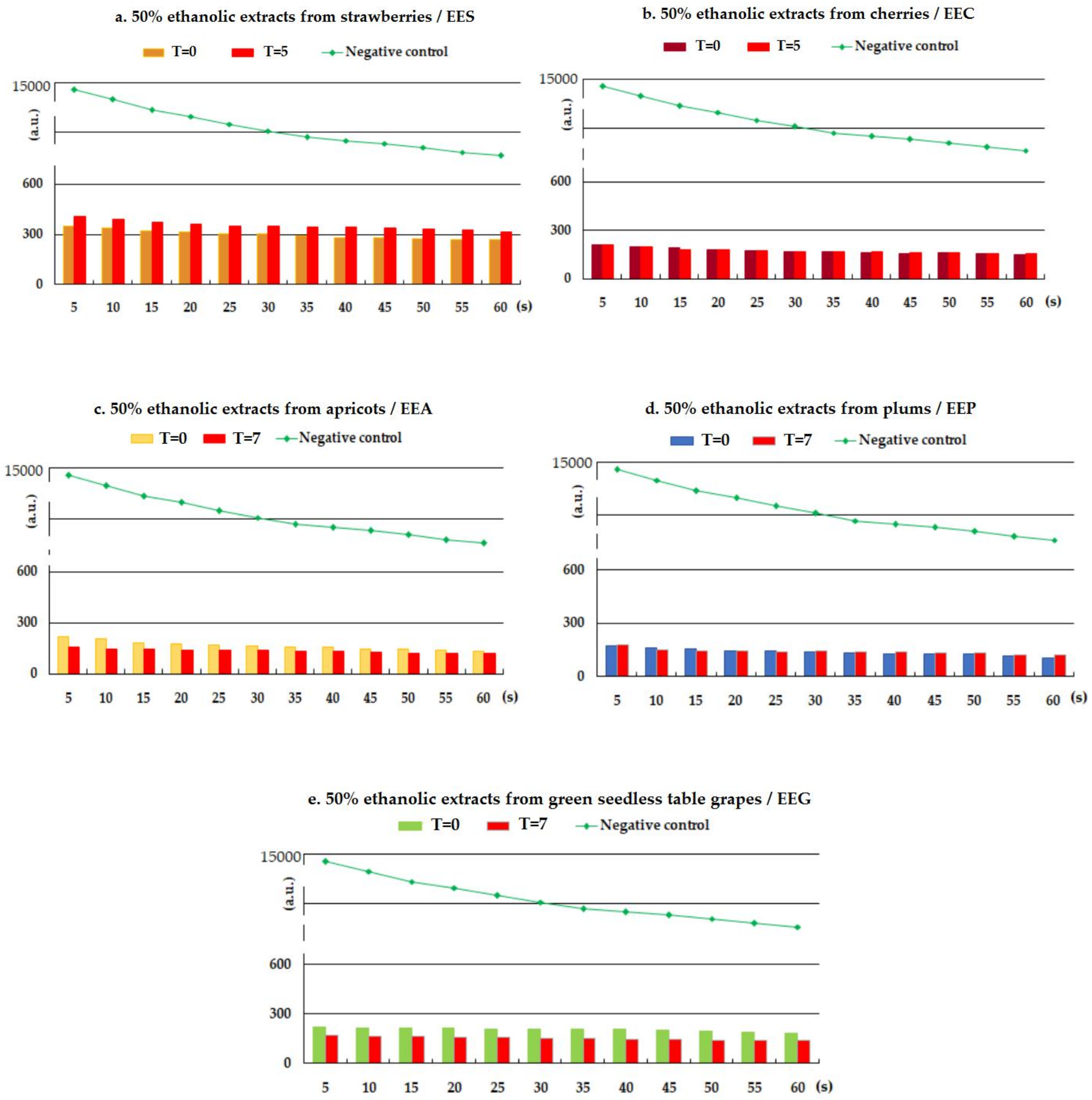

Figure S2. Technological aspects along the separation of the two test products from the five fruits series in the study; the acetone extracts further processed as 50% ethanolic extracts (EE) and the resulted fruit wastes namely acetone powders (AP) from strawberries, cherries, apricots, plums and green seedless table grapes.

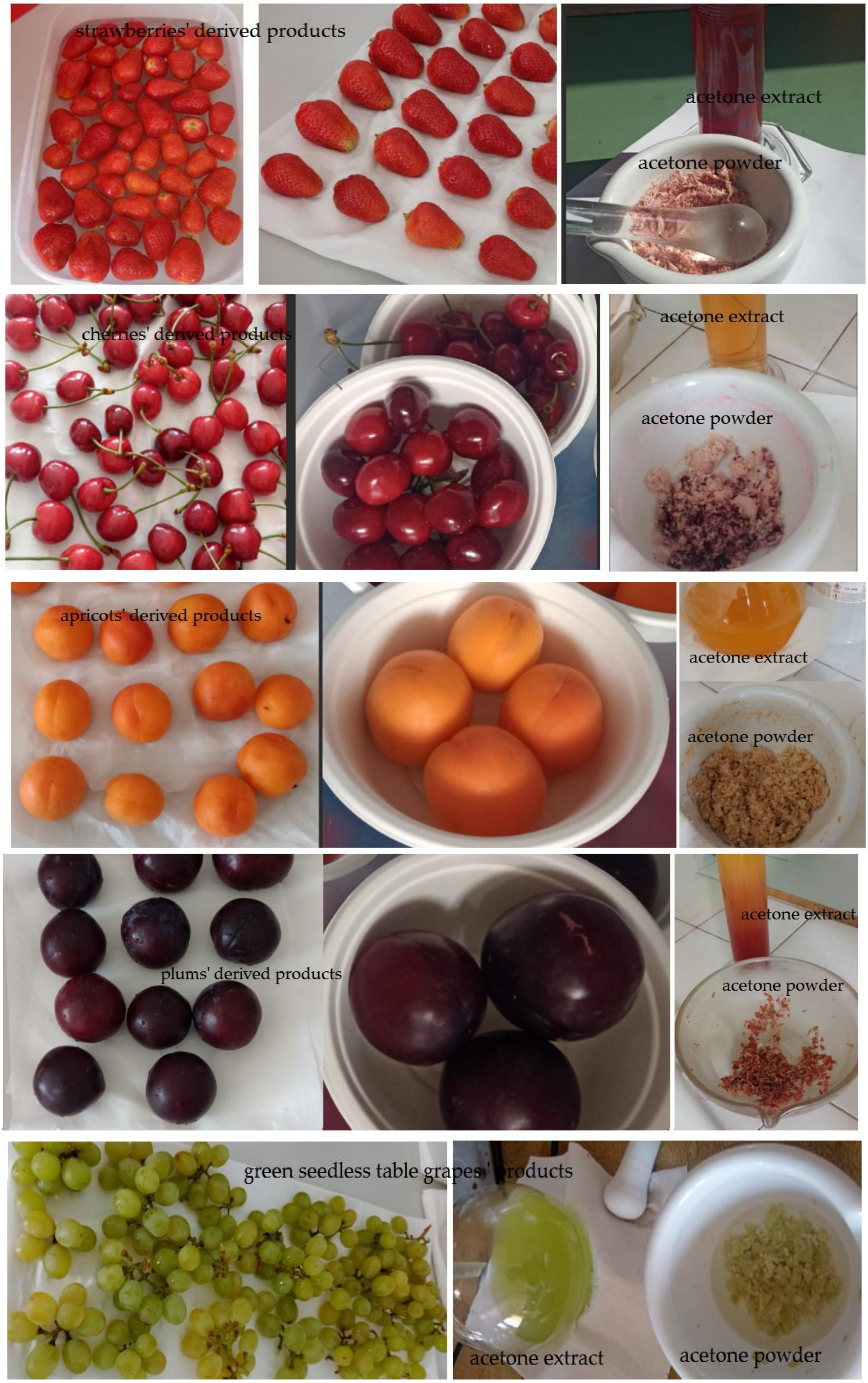

Supplement: Supplementary file 1 [file ijms-25-04848-s001.zip › ijms-2964258-supplementary.pdf]
